# Synergistic interaction between bedtime and eating speed in predicting overweight and obesity in Chinese preschool-aged children

**DOI:** 10.18632/aging.101906

**Published:** 2019-04-12

**Authors:** Shufang Liu, Jiachen Zhang, Jia Ma, Yu Shang, Yanyan Ma, Xinzhu Zhang, Shunan Wang, Yuan Yuan, Xiangling Deng, Wenquan Niu, Zhixin Zhang

**Affiliations:** 1 Beijing University of Chinese Medicine, Beijing, China; 2 Department of Pediatrics, China-Japan Friendship Hospital, Beijing, China; 3 Department of Pediatrics, Beijing Chaoyang District Maternal and Child Health Care Hospital, Beijing, China; 4 Beijing Hospital of Traditional Chinese Medicine, Beijing, China; 5 Institute of Clinical Medical Sciences, China-Japan Friendship Hospital, Beijing, China

**Keywords:** late bedtime, fast eating speed, overweight, obesity, preschool-aged children

## Abstract

This study aimed to examine the association of late bedtime and fast eating speed, both individually and interactively, in predicting overweight and obesity in Chinese preschool-aged children. This was a cross-sectional survey among children aged 3–6 years. Overweight and obesity is defined according to the WHO, IOTF, and China criteria, respectively. Total 1123 preschool-aged children were analyzed. After multivariable adjustment, late bedtime after 11:00 pm and fast eating speed increased the risk of overweight and obesity significantly under the WHO (odds ratio [OR]=1.92 and 1.37, 95% confidence interval [CI]: 1.31–2.80 and 1.00–1.88), IOTF (OR=1.47 and 1.46; 95% CI: 1.00–2.15 and 1.07–2.00), and China (OR=1.66 and 1.39; 95% CI: 1.20–2.29 and 1.07–1.80) criteria. Relative to bedtime before 11:00 pm and eating speed ≥30 min, there was a graded increase with presence of either bedtime after 11:00 pm or eating speed 15-30 min and <15 min. Particularly, the presence of both bedtime after 11:00 and eating speed <15 min yielded the largest OR under the WHO (adjusted OR, 95% CI: 3.98, 1.27–12.51), IOTF (3.59, 1.12–11.50), and China (4.84, 1.71–13.69) criteria. Taken together, our findings indicate a synergistic interaction between bedtime and eating speed in predicting overweight and obesity in Chinese preschool-aged children.

## INTRODUCTION

Childhood overweight and obesity has escalated to epidemic proportions worldwide, and it constitutes a major public health problem [1]. Global statistics show that the prevalence of overweight and obesity in preschool-aged children increases from 4.2% in 1990 to 6.7% in 2010, and it is projected to reach as high as 9.1% (approximately 60 million) in 2020 [2]. In China, the trend of childhood overweight and obesity had been rising dramatically during the past few decades, and a large survey shows that obesity prevalence increases from 0.91% in 1986 to 3.44% in 2006 [3]. Pediatric overweight and obesity may not only continue into adulthood [4], but be prone to metabolic abnormalities and pulmonary disorders [5–7]. Thus, efforts to control overweight and obesity in children must be pursued. The challenge ahead is the identification of possible risk factors responsible for childhood overweight and obesity. Although several epidemiological factors have been acknowledged for the development of obesity [8–11], no consensus has been reached yet. It is possible that obesity may be the resultant of the interaction between multiple factors, each of which carries a minor contribution.

There is wide recognition that bedtime and dietary speed are two key modifiable components in childhood weight gain [12–16]. There is evidence that preschool-aged children with early weekday bedtime were one-half as likely as children with late bedtime to be obese as adolescents [17], indicating that bedtime is a modifiable routine that may help to prevent obesity. In the medical literature, most studies define late bedtime as after 9:00 or 9:30 pm [18, 19], and further late bedtime has not yet been investigated. Besides late bedtime, unhealthy eating habits such as fast eating speed are widely studied as another possible risk factor for childhood obesity [20]. A national survey demonstrated that eating fast was positively associated with general and abdominal obesity among Chinese children [21]. Others proposed that changing eating speed may be an effective intervention for childhood obesity [22, 23]. Given that obesity development is a complex process, the effect of any risk factor may be small when assessed individually, but may be more pronounced in the presence of modifiable risk factors. A literature search has yet failed to reveal any evidence concerning the possible interaction between late bedtime and fast eating speed. To yield more information, we here developed a hypothesis that late bedtime and fast eating speed may act interactively in predisposition to childhood overweight and obesity.

To test this hypothesis, we conducted a cross-sectional survey in five kindergartens from Beijing to examine the association of late bedtime after 11:00 pm and fast eating speed, both individually and interactively, in predicting the risk of overweight and obesity in Chinese preschool-aged children.

## RESULTS

### Overweight and obesity prevalence

The present analysis involved a total of 1123 qualified preschool-aged children (mean age: 5.12 years old), including 559 boys and 564 girls. The prevalence of overweight and obesity as a whole was 15.12% (overweight: 8.9% and obesity: 7.03%) and 15.58% (overweight: 7.12% and obesity: 8.46%) under the World Health Organization (WHO) and International Obesity Task Force (IOTF) criteria, respectively. Under the China criteria, the prevalence of overweight and obesity was as high as 22.47% (overweight: 11.13% and obesity: 11.34%).

### Baseline characteristics

The baseline characteristics of study children are summarized in Table 1. The distributions of age, body mass index (BMI), maternal BMI, paternal BMI, pregnancy BMI, birth weight, daily sleep duration, bedtime, and eating speed differed significantly between the non-overweight group and the overweight and obesity group (all P<0.05) under three different growth criteria. Under the WHO and IOTF criteria, birth length differed significantly between the two groups, yet no significance was detected under the China children criteria. Sweet food and night meal intake frequency were comparable between the two groups. Fast food intake frequency showed no statistical significance under the WHO criteria and China criteria, and reached marginal significance under the IOTF criteria (P=0.045).

**Table 1:** The baseline characteristics of the non-overweight and the overweight and obesity groups under three different growth criteria.

**Variables**	**WHO criteria**			**IOTF criteria**			**China criteria**		
	**Non-overweight**	**Overweight/obesity**	**P**	**Non-overweight**	**Overweight/obesity**	**P**	**Non-overweight**	**Overweight/obesity**	**P**
Age									
≤5 years	488 (51.69%)	50 (27.93%)	<0.001	467 (49.26%)	71 (40.57%)	0.034	422 (48.79%)	116 (44.96%)	0.280
>5 years	456 (48.31%)	129 (72.07%)		481 (50.74%)	104 (59.43%)		443 (51.21%)	142 (55.04%)	
Males	457 (48.41%)	102 (56.98%)	0.035	469 (49.47%)	90 (51.43%)	0.634	410 (47.40%)	149 (57.75%)	0.004
BMI (kg/m^2^)	15 (14.18, 15.88)	20.11 (18.05, 28.12)	<0.001	15 (14.18, 15.88)	20.49 (18.18, 28.12)	<0.001	14.86 (14.12, 15.61)	18.20 (17.23, 23.93)	<0.001
Maternal BMI (kg/m^2^)	20.76 (19.43, 22.48)	21.81 (19.94, 23.87)	<0.001	20.76 (19.47, 22.48)	21.99 (19.92, 23.95)	<0.001	20.70 (19.30, 22.41)	21.71 (19.96, 23.59)	<0.001
Paternal BMI (kg/m^2^)	24.48 (22.60, 26.42)	25.25 (23.40, 26.87)	0.003	24.49 (22.60, 26.37)	25.62 (23.55, 27.47)	<0.001	24.39 (22.60, 26.28)	25.47 (23.51, 27.40)	<0.001
pregnancy BMI (kg/m^2^)	25.91 (24.09, 28.01)	26.62 (24.91, 28.51)	0.004	25.91 (24.09, 27.94)	26.70 (24.91, 28.91)	<0.001	25.81 (24.03, 27.94)	26.57 (25.10, 28.58)	<0.001
Birth length (cm)	50 (49, 51)	50 (50, 52)	0.034	50 (49, 51)	50 (50, 52)	0.011	50 (49, 51)	50 (50, 52)	0.151
Birth weight (×500 g)	6.8 (6.06, 7.38)	7 (6.4, 7.6)	0.009	6.8 (6.06, 7.36)	7 (6.4, 7.6)	<0.001	6.8 (6.02, 7.34)	7 (6.4, 7.6)	0.005
Daily sleep duration									
Less than 8 hours	17 (1.81%)	4 (2.25%)	0.007	16 (1.70%)	5 (2.87)	0.026	16 (1.86%)	5 (1.95%)	0.027
8–10 hours	767 (81.77%)	161 (90.45%)		774 (82.17%)	154 (88.51%)		701 (81.61%)	227 (83.15%)	
More than 10 hours	154 (16.42%)	13 (7.3%)		152 (16.14%)	15 (8.62%)		142 (16.53%)	167 (14.96%)	
Bedtime after 11:00 pm	347 (36.95%)	93 (51.96%)	<0.001	357 (37.86%)	83 (47.43%)	0.017	316 (36.74%)	124 (48.06%)	<0.001
Sweet food intake frequency									
Every day	167 (17.84%)	27 (15.08%)	0.644	170 (18.09%)	24 (13.71%)	0.354	152 (17.74%)	42 (16.28%)	0.767
Often (3-4 times weekly)	605 (64.64%)	118 (65.92%)		606 (64.47%)	117 (66.86%)		556 (64.88%)	167 (64.73%)	
None or once in a while	164 (17.52%)	34 (18.99%)		164 (17.45%)	34 (19.43%)		149 (17.395)	49 (18.99%)	
Fast food intake frequency									
Often (3-4 times weekly)	191 (20.30%)	46 (25.70%)	0.105	190 (20.11%)	47 (26.86%)	0.045	173 (20.07%)	64 (24.81%)	0.102
None or once in a while	750 (79.7%)	133 (74.30%)		755 (79.89%)	128 (73.14%)		689 (79.93%)	194 (75.19%)	
Night meal intake frequency									
Every day	106 (11.28%)	19 (10.61%)	0.228	109 (11.55%)	16 (9.14%)	0.254	94 (10.92%)	31 (12.02%)	0.439
Often (3–4 times weekly)	212 (22.55%)	51 (28.49%)		214 (22.67%)	49 (28%)		196 (22.76%)	67 (25.97%)	
None or once in a while	622 (66.17%)	109 (60.89%)		621 (65.78%)	110 (62.86%)		571 (66.32%)	160 (62.02%)	
Eating speed									
Less than 15 min	274 (29.15%)	78 (44.32%)	<0.001	277 (29.34%)	75 (43.6%)	0.001	252 (29.27%)	100 (39.22%)	0.002
15–30 min	543 (57.77%)	83 (47.16%)		542 (57.42%)	84 (48.84%)		491 (57.03%)	135 (52.94%)	
More than 30 min	123 (13.09%)	15 (8.52%)		125 (13.24%)	13 (7.56%)		118 (13.70%)	20 (7.84%)	

Abbreviations: BMI, body boss index; WHO, World Health Organization; IOTF, International Obesity Task Force.

Data are expressed as median (interquartile range) or count (percent). P value was calculated by the t-test or the rank-sum test or the Chi-squared test where appropriate.

### Identification of risk factors for overweight and obesity

Shown in Table 2 are the effect-size estimates of multiple examined factors in association with the risk of overweight and obesity before and after adjusting for confounding factors under three different growth criteria. Irrespective of adjustment for age and gender, the risk prediction of maternal BMI, paternal BMI, pregnancy BMI, birth weight, daily sleep duration, bedtime, and eating speed were significantly associated with the presence of overweight and obesity relative to the non-overweight group under the three criteria. After multivariable adjustment, only late bedtime after 11:00 pm and fast eating speed increased the risk of overweight and obesity significantly under the WHO (odds ratio [OR]=1.92 and 1.37, 95% confidence interval [CI]: 1.31–2.80 and 1.00–1.88, respectively), IOTF (OR=1.47 and 1.46; 95% CI: 1.00–2.15 and 1.07–2.00, respectively), and China (OR=1.66 and 1.39; 95% CI: 1.20–2.29 and 1.07–1.80, respectively) criteria. The power to detect the significant association between late bedtime after 11:00 pm and the risk of overweight and obesity was estimated to be 96.1%, 65.9%, and 89.9% under the WHO, IOTF, and China criteria, respectively. The corresponding power for fast eating speed was 84.6%, 84.8%, and 77.4%.

**Table 2:** The risk prediction for overweight and obesity in Chinese preschool-aged children.

**Variables**	**WHO criteria**			**IOTF criteria**			**China criteria**		
	**OR**	**95% CI**	**P**	**OR**	**95% CI**	**P**	**OR**	**95% CI**	**P**
***Unadjusted***									
Age	2.03	1.66–2.49	<0.001	1.51	1.24–1.84	<0.001	1.28	1.09–1.52	0.004
Gender	1.41	1.02–1.95	0.036	1.08	0.78–1.49	0.634	1.52	1.15–2.01	0.004
Maternal BMI	1.14	1.07–1.20	<0.001	1.15	1.09–1.22	<0.001	1.34	1.08–1.19	<0.001
Paternal BMI	1.08	1.03–1.15	0.003	1.12	1.06–1.18	<0.001	1.11	1.06–1.17	<0.001
Pregnancy BMI	1.08	1.02–1.14	0.010	1.11	1.04–1.17	0.001	1.09	1.04–1.14	0.001
Birth length	1.05	0.99–1.11	0.113	1.02	0.96–1.09	0.495	1.04	0.99–1.09	0.156
Birth weight	1.14	1.02–1.29	0.024	1.18	1.05–1.33	0.005	1.16	1.05–1.29	0.005
Daily sleep duration	1.56	1.12–2.18	0.008	1.56	1.11–2.18	0.010	1.38	1.02–1.85	0.034
Bedtime after 11:00 pm	1.84	1.33–2.55	<0.001	1.48	1.07–2.05	0.018	1.59	1.20–2.11	0.001
Sweet food intake frequency	0.89	0.68–1.16	0.382	0.83	0.64–1.10	0.193	0.92	0.72–1.16	0.467
Fast food intake frequency	1.36	0.94–1.97	0.106	1.46	1.01–2.11	0.045	1.31	0.95–1.82	0.103
Night meal intake frequency	1.10	0.88–1.34	0.410	1.01	0.80–1.28	0.926	1.12	0.92–1.36	0.268
Eating speed	1.66	1.28–2.17	<0.001	1.67	1.28–2.18	<0.001	1.50	1.19–1.86	0.001
***Age- and gender-adjusted***									
Maternal BMI	1.14	1.08–1.21	<0.001	1.15	1.09–1.22	<0.001	1.14	1.08–1.20	<0.001
Paternal BMI	1.08	1.02–1.14	0.008	1.11	1.05–1.18	<0.001	1.11	1.06–1.16	<0.001
Pregnancy BMI	1.09	1.03–1.15	0.003	1.11	1.05–1.18	<0.001	1.09	1.04–1.15	<0.001
Birth length	1.03	0.97–1.10	0.287	1.01	0.95–1.08	0.700	1.03	0.97–1.08	0.338
Birth weight	1.14	1.01–1.29	0.040	1.19	1.05–1.33	0.005	1.15	1.03–1.28	0.012
Daily sleep duration	1.36	0.96–1.94	0.083	1.43	1.01–2.02	0.042	1.32	0.97–1.79	0.078
Bedtime after 11:00 pm	1.79	1.29–2.49	0.001	1.44	1.04–2.00	0.029	1.57	1.18–2.08	0.002
Sweet food intake frequency	0.94	0.71–1.23	0.639	0.85	0.65–1.12	0.261	0.95	0.75–1.20	0.677
Fast food intake frequency	1.33	0.91–1.95	0.141	1.43	0.99–2.08	0.059	1.30	0.93–1.81	0.120
Night meal intake frequency	1.13	0.89–1.42	0.304	1.02	0.81–1.30	0.840	1.13	0.93–1.38	0.220
Eating speed	1.53	1.16–2.02	0.002	1.47	1.23–2.12	<0.001	1.43	1.14–1.80	0.002
***Multivariable adjusted***									
Maternal BMI	1.08	0.99–1.18	0.094	1.08	0.99–1.18	0.081	1.09	1.01–1.17	0.031
Paternal BMI	1.05	0.99–1.12	0.128	1.09	1.02–1.17	0.008	1.09	1.03–1.15	0.002
Pregnancy BMI	1.06	0.99–1.15	0.135	1.08	0.99–1.17	0.072	1.05	0.98–1.12	0.174
Birth length	0.99	0.92–1.07	0.884	0.97	0.91–1.04	0.422	0.99	0.94–1.06	0.837
Birth weight	1.12	0.97–1.30	0.123	1.19	1.04–1.36	0.014	1.13	1.00–1.28	0.051
Daily sleep duration	1.21	0.79–1.87	0.385	1.23	0.80–1.91	0.349	1.16	0.80–1.68	0.446
Bedtime after 11:00 pm	1.92	1.31–2.80	0.001	1.47	1.00–2.15	0.049	1.66	1.20–2.29	0.002
Sweet food intake frequency	0.84	0.61–1.18	0.316	0.78	0.56–1.09	0.140	0.88	0.66–1.16	0.370
Fast food intake frequency	1.38	0.88–2.16	0.165	1.63	1.04–2.54	0.033	1.31	0.89–1.93	0.169
Night meal intake frequency	0.98	0.73–1.31	0.879	0.90	0.66–1.21	0.478	1.01	0.79–1.30	0.914
Eating speed	1.37	1.00–1.88	0.048	1.46	1.07–2.00	0.018	1.39	1.07–1.80	0.014

Abbreviations: BMI, body boss index; WHO, World Health Organization; IOTF, International Obesity Task Force; OR, odds ratio; 95% CI, 95% confidence interval. P values were calculated before and after adjusting for age, gender, maternal BMI, paternal BMI, pregnancy BMI, birth length, birth weight, dietary and sleep habits. In multivariable adjusted model, risk prediction of each adjusted factor was calculated by adjusting for the other factors.

### Interaction between bedtime and eating speed

In view of individual significance for bedtime and eating speed, further explorations on their potential interaction were conducted under three different criteria (Table 3). Six combinations were generated under the two categories of bedtime and the three categories of eating speed. Using bedtime before 11:00 pm and eating speed ≥30 min as a reference combination, there was a graded increase with the single presence of either bedtime after 11:00 pm or eating speed 15-30 min and <15 min. In particular, the presence of both bedtime after 11:00 and eating speed <15 min yielded the largest effect-size estimate under the WHO criteria (OR=5.74 and 3.98, 95% CI: 2.14–15.40 and 1.27–12.51), IOTF criteria (OR=4.89 and 3.59, 95% CI: 1.81–13.17 and 1.12–11.50), and China criteria (OR=6.12 and 4.84, 95% CI: 2.46–15.23 and 1.71–13.96) before and after adjusting for confounding factors, indicating a potential synergistic interaction between bedtime and eating speed.

**Table 3:** The interaction between bedtime and eating speed in predicting overweight and obesity in Chinese preschool-aged children.

**Sleep/eating speed**	**WHO criteria**			**IOTF criteria**			**China criteria**		
	**OR**	**95% CI**	**P**	**OR**	**95% CI**	**P**	**OR**	**95% CI**	**P**
***Unadjusted***									
Bedtime before 11:00 pm/eating speed ≥30 min	Reference			Reference			Reference		
Bedtime before 11:00 pm/eating speed 15-30 min	1.55	0.59–4.11	0.369	1.80	0.69–4.70	0.233	2.64	1.10–6.32	0.029
Bedtime before 11:00 pm/eating speed <15 min	3.28	1.24–8.62	0.016	3.37	1.28–8.86	0.014	3.98	1.64–9.66	0.002
Bedtime after 11:00 pm/eating speed ≥30 min	2.73	0.88–8.47	0.082	2.10	0.65–6.79	0.214	3.40	1.22–9.48	0.019
Bedtime after 11:00 pm/eating speed 15-30 min	3.17	1.21–8.29	0.019	2.84	1.08–7.46	0.034	4.13	1.72–9.98	0.002
Bedtime after 11:00 pm/eating speed <15 min	5.74	2.14–15.40	0.001	4.89	1.81–13.17	0.002	6.12	2.46–15.23	<0.001
Multivariable adjusted^*^									
Bedtime before 11:00 pm/eating speed ≥30 min	Reference			Reference			Reference		
Bedtime before 11:00 pm/eating speed 15-30 min	1.40	0.46–4.27	0.551	1.75	0.57–5.40	0.328	2.42	0.90–6.56	0.081
Bedtime before 11:00 pm/eating speed <15 min	2.31	0.76–7.05	0.142	2.83	0.91–8.77	0.072	3.43	1.25–9.38	0.017
Bedtime after 11:00 pm/eating speed ≥30 min	4.07	1.13–14.61	0.031	3.05	0.81–11.54	0.101	4.19	1.32–13.34	0.015
Bedtime after 11:00 pm/eating speed 15-30 min	2.35	0.78–7.17	0.131	2.29	0.74–7.12	0.152	3.59	1.32–9.81	0.013
Bedtime after 11:00 pm/eating speed <15 min	3.98	1.27–12.51	0.018	3.59	1.12–11.50	0.032	4.84	1.71–13.69	0.003

Abbreviations: WHO, World Health Organization; IOTF, International Obesity Task Force; OR, odds ratio; 95% CI, 95% confidence interval. P values were calculated before and after* adjusting for age, gender, maternal body boss index (BMI), paternal BMI, pregnancy BMI, birth length, birth weight, other dietary and sleep habits.

### Prediction model

**Figure 1:**
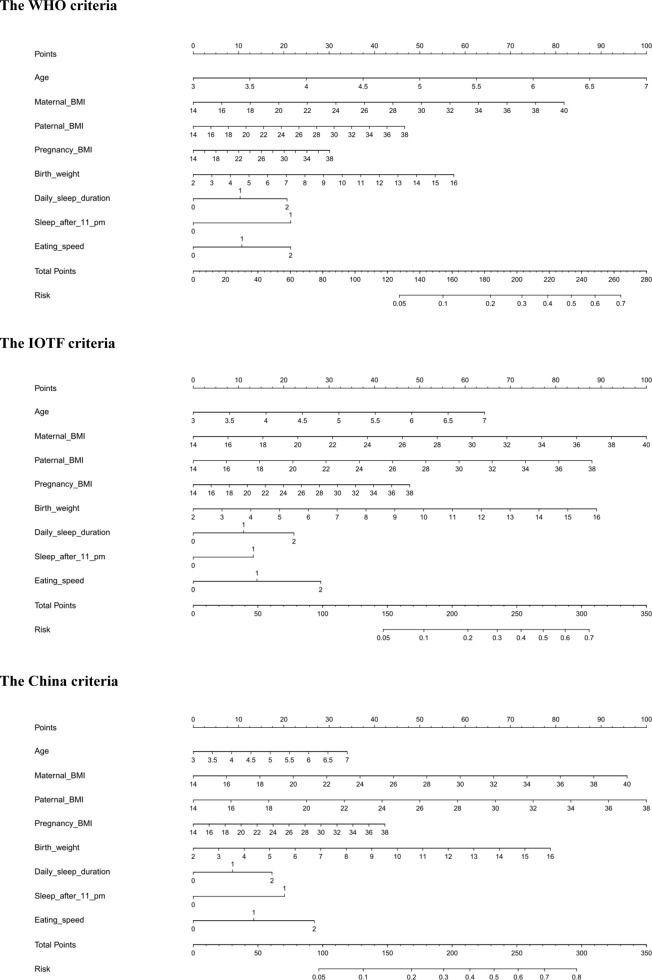
**The prediction nomograms of significant factors for the risk of overweight and obesity under the WHO (the upper panel), IOTF (the middle panel) and China (the lower panel) criteria.** Abbreviations: WHO, World Health Organization; IOTF, International Obesity Task Force.

Finally, a nomogram model to predict the risk of overweight and obesity was constructed based on significant factors under three different criteria, as displayed in Figure 1. Significant factors in nomogram model included age, maternal BMI, paternal BMI, pregnancy BMI, birth weight, daily sleep duration, bedtime, and eating speed, which were selected by forward Logistic regression analyses at a significance level of 5%. Taking the nomogram model under the WHO criteria as an example, assuming a child aged 5 years old (50 points) with maternal BMI of 30 kg/m^2^ (50 points), paternal BMI of 28 kg/m^2^ (37 points), pregnancy BMI of 34 kg/m^2^ (25 points), birth weight of 4 kg (25 points), sleep duration less than 8 hours (20 points), sleeping after 11:00 pm (20 points), and eating speed <15 min (20 points), the probability of overweight and obesity was estimated to be 63%.

The predictive accuracy of three nomogram models was good, and the C-index was 0.718, 0.688, and 0.663 respectively under the WHO, IOTF, and China criteria (all P<0.001).

## DISCUSSION

The aim of this cross-sectional study was to examine the association of late bedtime after 11:00 pm and fast eating speed, both individually and interactively, in predicting the risk of overweight and obesity among 1123 Chinese preschool-aged children. The most noteworthy finding of this study is the synergistic interaction between bedtime and eating speed in predicting overweight and obesity under three different growth criteria. To the best of our knowledge, this is thus far the first report that has interrogated the bedtime-eating speed interaction in the medical literature. Our findings highlight the importance of propagating early bedtime and slow eating speed in preschool-aged children to control or prevent the development of overweight and obesity.

In the process of childhood development and growth, sleep and eating plays a key role in weight management [24]. Although some studies showed that short sleep duration was a risk factor for childhood overweight and obesity [13, 25, 26], sleep duration is closely associated with bedtime and weak time. There is evidence that the impact of bedtime for sleep duration was greater than weak time [14, 27]. In particular, the nationally representative data of American children aged 3–18 years showed that in younger children, predicted standardized BMI increased linearly with the increase of bedtime from 7:30 pm to 11:30 pm [27]. Similarly, results of a cohort study indicated that irregular bedtime was a strong predictor for increased BMI in children [28]. Our findings indicated that bedtime after 11:00 pm was 1.47 to 1.92 times more likely to be overweight or obese in preschool-aged children. Hence, it is important to foster a good sleep habit to maintain childhood healthy growth.

Moreover, our findings also showed the significant association of eating speed with overweight and obesity, in agreement with the results of some previous studies [21, 29, 30]. As indicated in a Singapore study, children who ate fast was associated with an increased BMI z-score and adiposity (4.5 years old) [29]. In a Japan study, stopping a quick eating habit can prevent excess gains in anthropometric indexes among non-overweight/obese girls, and further contribute to the prevention of childhood overweight and obesity [30]. Importantly, results of a randomized clinical trial demonstrated that slower eating might be a novel target for family-based obesity prevention targeting high-risk children [23]. Based on above evidence, the habit of eating slowly is beneficial to physical health in children.

As an extension of prior studies, we interestingly observed a synergistic interaction between late bedtime and fast eating speed in Chinese preschool-aged children. Specifically, children with late bedtime after 11:00 pm and eating speed less than 15 min were approximately four times more likely to become overweight or obese. There is indirect epidemiological and clinical evidence for the mechanisms behind the interaction between bedtime and eating speed. The superimposition of excessive energy intake can be a possible explanation. On one hand, late bedtime was found to be related to low diet quality [31]. For instance, later sleepers were significantly associated with snacking in primary school children [19]. In return, children who consumed energy drinks exhibited an increased odds of having late bedtime compared with con-consumers [32]. On the other hand, the effect of eating speed on overweight and obesity in preschool-aged children is attributed to higher energy intake [29, 33] due to the interference of gastrointestinal hormones, such as ghrelin suppression. As evidenced, slower eating rate can be accomplished with greater fullness, greater ghrelin suppression post-meal, and less energy from snacks [22], and have a significant impact on gastrointestinal hormone response to a carbohydrate in retraining obese adolescents [34]. Despite the above potential explanations, the exact mechanism of the interaction between late bedtime and fast eating speed needs to be further elucidated.

What’s more, in view of significant predictive capability of bedtime and eating speed, we constructed three predictive nomogram models by incorporating all significant factors under three different growth criteria, which can be used in routine practice to provide more accurate prediction of being overweight or obese in preschool-aged children.

The clear strengths of this present study include the identification of a synergistic interaction between bedtime and eating speed, comparison of different growth criteria, and construction of an efficient prediction model. However, our study has some possible limitations. Firstly, the cross-sectional nature of our analyses limits the assumptions of any temporal relationship, which means that our data cannot be used to prove the existence of a cause-effect relationship between bedtime/eating speed and the development of childhood overweight and obesity. Secondly, anthropometric measures were obtained from questionnaires filled by child guardians, which might yield a recall bias. Thirdly, most previous studies defined late bedtime after 9:00 or 9:30 pm, yet in this present study late bedtime is defined after 11:00 pm. It is of added interest to compare two cutoff points for late bedtime. Fourthly, other information such as maternal pre-pregnancy obesity, time interval between dinner and bedtime was not recorded in this survey, and these factors are closely related to bedtime and eating habits, as well as their interaction. Nonetheless, we agree that more well-designed longitudinal studies are needed to confirm or refute the findings of this association study.

Despite the above limitations in the present study, our findings indicate a synergistic interaction between bedtime and eating speed in predicting overweight and obesity in Chinese preschool-aged children. This study might help to motivate parents and kindergarten teachers to increase awareness on the detrimental impact of late bedtime and fast eating speed, and the control and prevention of childhood overweight and obesity through effective strategies should be a priority.

## METHODS

### Study children

This was a cross-sectional survey of overweight and obesity among children aged 3 to 6 years in Chaoyang District, Beijing. This study was approved by the Ethics Committee of China-Japan Friendship Hospital, and informed consent was obtained from guardians of all children prior to participation.

Using a stratified cluster random sampling strategy, 1333 preschool-aged children from 5 kindergartens of Chaoyang District, Beijing were selected in this study, and a self-designed questionnaire was circulated to every child guardian from March to May in 2017. We collected 1327 questionnaires, and after excluding questionnaires with invalid gender, body height, and weight, there were 1123 validated questionnaires in the final analysis.

### Data collection and quality control

Questionnaire data included individual data of children and parents, and children dietary and sleep habits. Children individual data contained gender, age (year and month in details), height (to the nearest 0.1 cm), weight (to the nearest 0.1 kg), birth length, and birth weight. Body height and weight of parents and maternal pregnancy were also recorded. Children dietary and sleep habits were asked, including weekly frequency of sweet food, fast food and night meal consumption, eating speed, daily sleep duration and bedtime.

Before questionnaire circulation, each health physician from 5 kindergartens received the same training on investigation methods. Questionnaires were handed out and collected by kindergarten teachers, and were further checked by health physicians. Questionnaires were double checked by two authors (J.M. and S.W.), and missing information was complemented via telephone interview.

### Overweight and obesity definition

Overweight and obesity is classified by BMI, which is calculated as the ratio of both weight (kg) to the square of body height (m). Some criteria have been widely adopted to define overweight and obesity in preschool-aged children, and we employed the WHO criteria (2006), the IOTF criteria (2000) and the China criteria (2009) simultaneously in this present study.

Under the WHO criteria, there are two growth standards before and after 5 years old, using BMI z-scores to define overweight and obesity in our study. Before 5 years old, overweight and obesity are respectively defined as BMI z-scores >2 standard deviation (SD) and BMI z-score >3 SD [35]. After 5 years old, overweight and obesity are respectively defined as BMI z-score >1 SD and BMI z-score >2 SD [36].

Under the IOTF criteria, overweight and obesity are respectively defined to pass through BMI of 25 kg/m^2^ and 30 kg/m^2^ at age 18 [37].

Under the China criteria, overweight and obesity are respectively defined to pass through BMI of 24 kg/m^2^ and 28 kg/m^2^ at age 18 [38].

### Dietary and sleep definition

Dietary data included weekly frequency of sweet food, fast food and night meal intake, and eating speed. Sweet food is defined as food with high sugar (e.g. cake, sugar, dessert, and chocolate), and weekly intake frequency is sorted in every day, that is, often (3 to 4 times), none or once in a while. Fast food is defined as food with high energy and low nutrition (e.g. hamburger and French fries), weekly intake frequency is classified as often (3 to 4 times), and none or once in a while. Night meal is the same as the extra meal in a day, defined as eating some food within 2 hours before bedtime, and weekly intake frequency is consistent with sweet food. Eating speed is divided into fast (<15 min), moderate (15-30 min), and slow (≥30 min).

Sleep data contained sleep duration and bedtime. Sleep duration is classified as short (<8 hours), moderate (8-10 hours), and long (≥10 hours). Bedtime is categorized as bedtime before and after 11:00 pm.

### Statistical analysis

All preschool-aged children were classified into two groups — the non-overweight group and the overweight and obesity group, according to three growth criteria mentioned above. Categorical and continuous variables were respectively described using count (percent) and median (interquartile range), and they were compared between two groups by using the Chi-squared test, t-test and rank-sum test where appropriate. Logistic regression analyses were adopted to identify significant risk factors associated with overweight or obesity before and after adjusting for age, gender, maternal BMI, paternal BMI, pregnancy BMI, birth length, birth weight, dietary and sleep habits. Effect-size estimates are expressed as OR and 95% CI.

To facilitate clinical explanation, a prediction nomogram was created using significant risk factors under three different growth criteria, and predictive accuracy was determined by concordance index (C-index) and defined as the area under the receiver operating characteristics curve.

Study power was estimated using the PS-Power Simple Size software (version 3.1.2). The R-language (version 3.5.2) was adopted to generate nomogram. Statistical analyses were conducted using the STATA software special edition (version 14.0, Stata Corp, TX). P value less than 0.05 was considered as statistically significant.
